# Vertebroplasty: Patient and treatment variations studied through parametric computational models^☆^

**DOI:** 10.1016/j.clinbiomech.2013.07.012

**Published:** 2013-10

**Authors:** Vithanage N. Wijayathunga, Robert J. Oakland, Alison C. Jones, Richard M. Hall, Ruth K. Wilcox

**Affiliations:** Institute of Medical & Biological Engineering, Department of Mechanical Engineering, University of Leeds, UK

**Keywords:** Vertebroplasty, Finite element analysis, Adjacent vertebral failure, Degenerated intervertebral discs, Cement volume

## Abstract

**Background:**

Vertebroplasty is increasingly used in the treatment of vertebral compression fractures. However there are concerns that this intervention may lead to further fractures in the adjacent vertebral segments. This study was designed to parametrically assess the influence of both treatment factors (cement volume and number of augmentations), and patient factors (bone and disc quality) on the biomechanical effects of vertebroplasty.

**Methods:**

Specimen-specific finite element models of two experimentally-tested human three-vertebral-segments were developed from CT-scan data. Cement augmentation at one and two levels was represented in the respective models and good agreement in the predicted stiffness was found compared to the corresponding experimental specimens. Parametric variations of key variables associated with the procedure were then studied.

**Findings:**

The segmental stiffness increased with disc degeneration, with increasing bone quality and to a lesser extent with increasing cement volume. Cement modulus did not have a great influence on the overall segmental stiffness and on the change in the elemental stress in the adjoining vertebrae. However, following augmentation, the stress distribution in the adjacent vertebra changed, indicating possible load redistribution effects of vertebroplasty.

**Interpretation:**

This study demonstrates the importance of patient factors in the outcomes of vertebroplasty and suggests that these may be one reason for the variation in clinical results.

## 1 Introduction

The estimated annual incidence of vertebral compression fractures in Europe is nearly 1.4 million (Anon, 2002), many of which lead to severe pain and a significant reduction in quality of life. There has been a rise in the use of vertebroplasty for the treatment of these fractures over the last decade. Notwithstanding differences in study designs, the results of recent randomised controlled trials have reported inconsistent patient outcomes and contrastingly different conclusions (Buchbinder et al., 2009; Kallmes et al., 2009; Klazen et al., 2010). One possibility for these differing results could be that, from the general cohort of back-pain patients identified for routine vertebroplasty, some subgroups gain more benefit from the procedure than others. This raises questions about the current understanding of the biomechanics of the procedure as well as broader issues of pain management and its relationship to biomechanical function. There is also debate as to whether the treatment may predispose patients to additional fractures in the adjacent vertebrae with a number of retrospective studies suggesting an association between vertebroplasty and new-onset adjacent fractures (Kim et al., 2004; Komemushi et al., 2006). However, it is still unclear whether these fractures are a direct result of the treatment, additional local kyphosis following fracture or the natural progression of osteoporosis with weakening of the vertebral bone.

Laboratory models have reported differing effects of vertebroplasty on the adjacent vertebral behaviour. Berlemann et al. (2002) found lower failure strengths on augmented segments compared to BMD-matched non-augmented segments, whilst Boger et al. (2007) found no significant difference in strength. Several computational studies have indicated an alteration in the stress distribution of augmented and adjacent vertebrae following vertebroplasty (Baroud et al., 2003; Polikeit et al., 2003; Wilcox, 2006) due to the higher stiffness of the cement than the surrounding bone. However, Villarraga et al. (2005) in a study of kyphoplasty found that changes in the adjacent vertebral strains were minimal. Further, Rohlmann et al. (2005) concluded that an increased flexion moment due to the wedge fracture would have a greater effect on intra-discal pressure and the adjacent vertebral behaviour than the cement-augmentation.

It is not apparent whether the lack of consistency in the outcomes of these studies is due to differences in experimental or computational set-up, such as the method of load application and boundary conditions, or due to differences in the underlying specimens or parameters used in the model construction. The relationship between adjacent fracture risk and patient factors such as the bone and intervertebral disc condition or treatment factors such as the injected cement volume has not been fully investigated. Therefore, the aim of this study was to parametrically assess the influence of both patient and clinical variables on the biomechanical effects of vertebroplasty. A specimen-specific finite element (FE) model was used, allowing the baseline results to be validated against corresponding experimental data.

## 2 Methods

### 2.1 Experimental methods

Following Research Ethics Committee approval (No. 06/Q1206/149), two human three-vertebra (T12-L1-L2) segments (female, > 70 years) were selected from two related experimental studies reported previously (Oakland et al., 2008, 2009). Specimen 1 was selected to generate the finite element model used in the parametric study, and was used to derive the degenerated disc properties. This specimen was augmented in a prophylactic manner with vertebroplasty taking place at the L1 level. Specimen 2 had augmentations in T12 and L1 levels, and was used to support the model validation.

Briefly, the experimental protocol involved the following steps. The specimens were housed in cement fixtures. Imaging was undertaken by quantitative computed tomography (QCT) (PQ-2000, Picker, US, spatial resolution = 2 mm, 65 mA, 140 kV), using a hydroxyapatite phantom for bone mineral density (BMD) calibration. A compressive load up to 50% of the predicted failure of L1was applied via a steel ball through the central axis of the specimen at 1 mm/min, in a material testing machine (AGS-10kNG, Shimadzu, Kyoto, Japan) and the stiffness determined from the average load-displacement gradient over a 0.6 mm range from three loading cycles. The cement augmentation was with Vertebroplastic cement (DePuy, Leeds, UK) until an estimated fill of 20% of vertebral body volume was achieved.

### 2.2 Computational methods

**Fig. 1:**
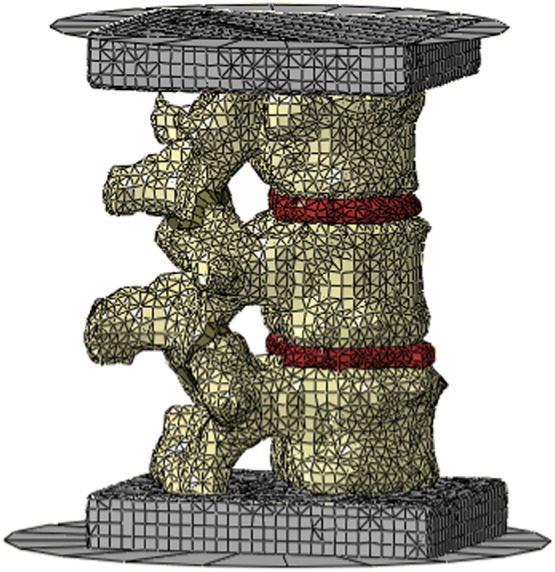
The completed finite element model for Specimen 1 showing the three-vertebral segment potted in cement at either end and attached to rigid plates to represent the housing used in the experimental testing.

Proprietary software (ScanIP, Simpleware, UK) was used to segment the CT-images and develop FE meshes for both specimens (Fig. 1). In order to identify the vertebrae, the intervertebral discs and the cement housings from the scanned data, a series of image processing steps were undertaken. These included greyscale thresholding to identify the constituent geometries according to their greyscale values, plus a combination of flood-fill, morphological closing and level set operations to maintain the geometric integrity of the components. This method has been previously described by Wijayathunga et al. (2008). Based on observations of the images, the disc tissue was manually segmented into nucleus and annulus regions. Generated volumetric FE mesh consisted of mixed hexahedral and tetrahedral elements. Following a previous convergence study (Jones and Wilcox, 2007) an approximate hexahedral element size of 1.4 mm was used for the trabecular-inner region of the vertebral body, and to maintain the specimen specific morphological characteristics, the outer cortical region of the vertebrae had tetrahedral elements of much smaller size. Vertebral models developed in this manner had been experimentally validated in a previous study (Wijayathunga et al., 2008).

An initial model of Specimen 1 was generated from the pre-treatment CT scan of the specimen. Models of both specimens representing the augmented cases were then generated using the post-treatment CT image data to identify cement regions to match with the experimental specimens.

The material properties of the model components are shown in Table 1. The elastic modulus values for the bone were assigned using the image greyscale data, following a method developed previously (Wijayathunga et al., 2008).

The intervertebral discs in both the experimental specimens were observed to be degenerated with narrowed disc space and evidence of osteophytes. Since no method currently exists for deriving the disc properties from the image data, the following approach was used. For the model of Specimen 1, the annulus and nucleus tissues were assigned with orthotropic and isotropic material models respectively, initially using material properties for healthy tissue obtained from literature [Table 1.]. These values were then sequentially altered, maintaining the same ratios of anisotropy for the annulus until the simulated segmental stiffness of the model matched that obtained from the pre-augmentation experimental test for Specimen 1. These derived degenerated disc properties were then also applied without further modification to the model of Specimen 2.

Sliding contact was defined in the facet joints with a friction coefficient of 0.1 (Polikeit et al., 2003). No other constraints were defined at the joint. The capsular ligament, represented by a series of membrane elements spanning the facet capsule, was included to maintain the physiological integrity of the joint during loading; however a sensitivity study showed that the presence of the ligament had minimum effect on the segmental behaviour under the loading regime applied in this case. Other ligaments were not included since the model was tested under axial compression only.

To replicate the experimental tests, the cement housings were included in the model and a tied constraint was used between the cement housings and the rigid plates to represent the steel loading platens in the testing machine. An axial displacement was applied to the mid-point of the anterior-posterior diameter of the top plate.

#### 2.2.1 Validation

The models were initially set up to represent the respective experimental specimens, with the same levels augmented as in the experiment (the ‘base state’). The pre-augmented model of Specimen 1 had already been used to tune the disc properties, so this model was not used for validation. However, since no further adjustments were made to the material properties of either model, the predicted stiffness values of both of the post-augmentation models were compared with the results obtained from the corresponding experimental tests.

#### 2.2.2 Parametric studies

A series of parametric studies were conducted where specific parameters were varied in turn from their base state values. In all cases, the overall model stiffness values were determined and compared. Where applicable, the change in the stress distribution in the adjacent vertebrae was also evaluated by comparing the von Mises stress in each element of the augmented model to the value in the same element in the pre-treatment model. Since the models were not validated in terms of stress, only the relative changes in stress distribution were examined, rather than the magnitudes.

The properties of the bone-cement composite have been shown to be lower than that of the pure cement (Boger et al., 2007; Race et al., 2007; Zhao et al., 2010). The modulus will vary depending on the underlying bone structure and the cement penetration characteristics. To simulate across the range of likely values reported in the literature, the elastic modulus of the cement-augmented region was varied from that of pure cement (2.04 GPa) to 50%, 25% and 12.5% of that value. In addition to the overall model stiffness, the corresponding changes in the von Mises stress distribution in the T12 and L2 vertebrae were also examined.

To examine the effect of the amount of cement injected, the volume of cement in L1-augmentation was doubled and halved by assigning a larger or smaller number of elements to the augmented region. When representing the smaller volume of cement, the remaining elements were re-assigned with their underlying bone properties.

The bone quality was varied by approximately halving and doubling the element-specific elastic modulus values from their base state to represent lower and higher bone density conditions respectively. The state of the intervertebral disc was varied between the degenerated base case and that of healthy tissue, using values taken from the literature for the latter as indicated above.

Finally, to examine the effect of spinal morphology and augmentation of multiple segments, two further scenarios were examined where the T12 vertebra was augmented instead of L1, and where both T12 and L1 vertebrae were augmented.

## 3 Results

### 3.1 Validation

Following sequential tuning of the disc properties of the model representing Specimen 1 to match the experimentally measured stiffness prior to cement augmentation (617 N/mm), the post-augmentation predicted stiffness (660 N/mm) was less than 1% different from the experimental value (665 N/mm). For the model representing Specimen 2, where the same disc properties were applied without further tuning, a post-augmentation error of 6% was found between the model (852 N/mm) and the experiment (806 N/mm).

### 3.2 Parametric studies

**Fig. 2:**
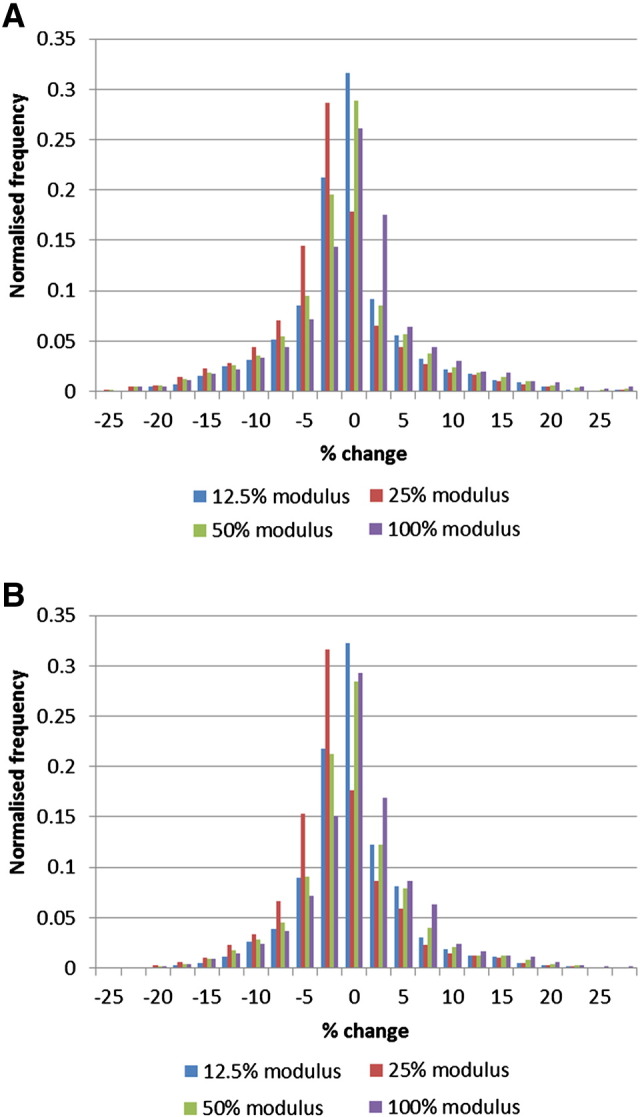
Distribution of the change in the von Mises stress in the T12 (Fig. 2A) and L2 (Fig. 2B) vertebrae when the cement modulus of the L1 augmentation is varied. The change is calculated as a percentage difference in stress in each element compared to the same element in the untreated model. Normalising is by the total number of elements in the corresponding vertebra.

The influence of the cement properties assigned to the augmented region on the overall segmental stiffness was found to be minimal, with changes of 0.7, 1.8 and 3.3% when the augmented region modulus was reduced by 50, 75 and 87.5% respectively. When the stress distributions in the adjacent vertebrae were compared to the non-augmented model, there were generally only small changes, as shown in Fig. 2. The differences between the models with different augmented region modulus values were also small, with only a slight shift towards more elements experiencing lower stresses as the augmentation modulus was reduced.

**Fig. 3:**
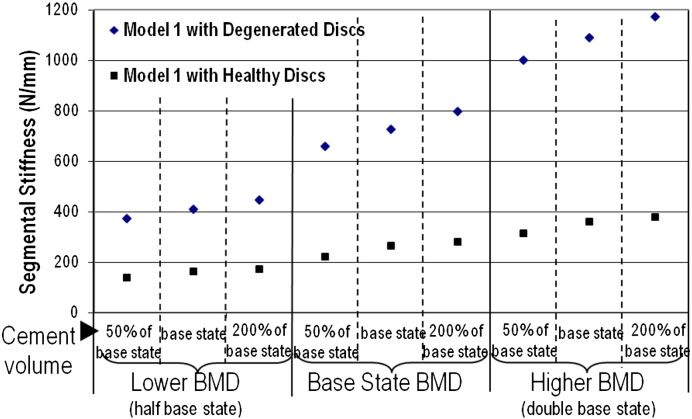
The segmental stiffness derived from Model 1 for parametric variations in bone density and cement augmentation volume.

The influence of the cement volume and patient variables on the segmental stiffness is summarised in Fig. 3. It was found that the stiffness increased considerably when the augmentation was carried out in a setting where the intervertebral discs were degenerated. The disc condition was found to have a greater influence on the segmental stiffness than the cement volume.

**Fig. 4:**
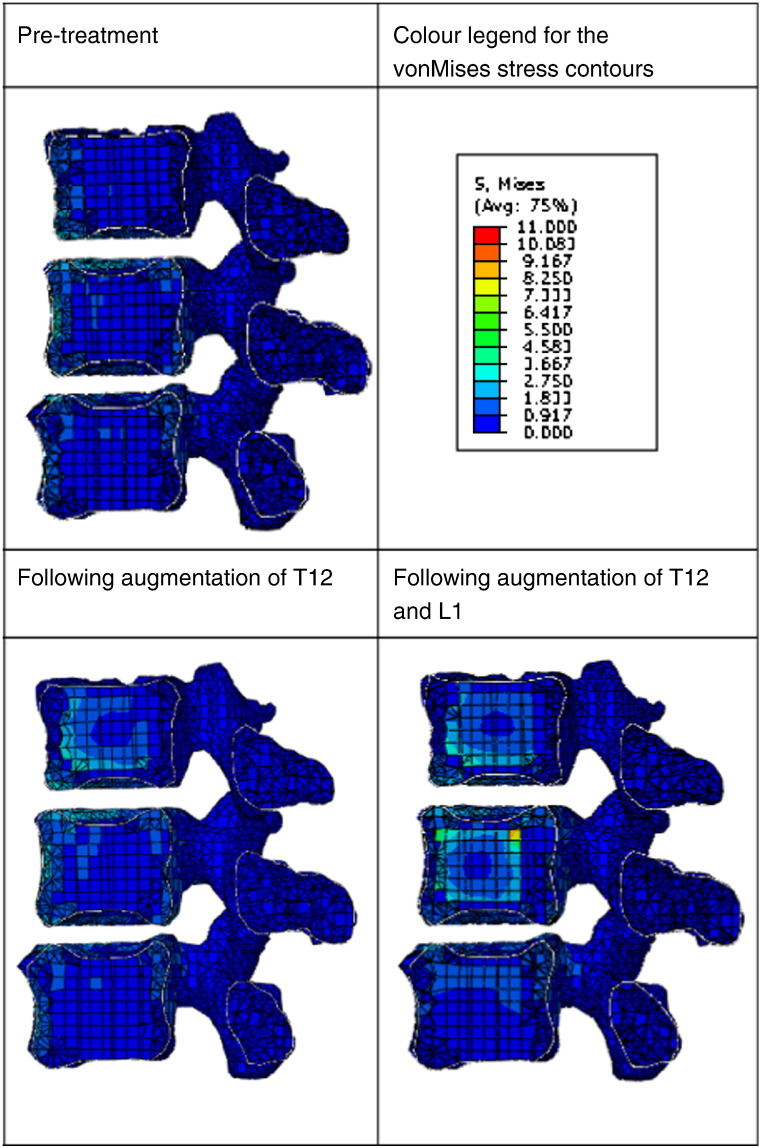
Cross sections of the finite element models pre- and post- augmentation of the T12 and L1 vertebrae showing the von Mises stress contours under a 1000 N load. The soft tissue components have been removed for clarity.

**Fig. 5:**
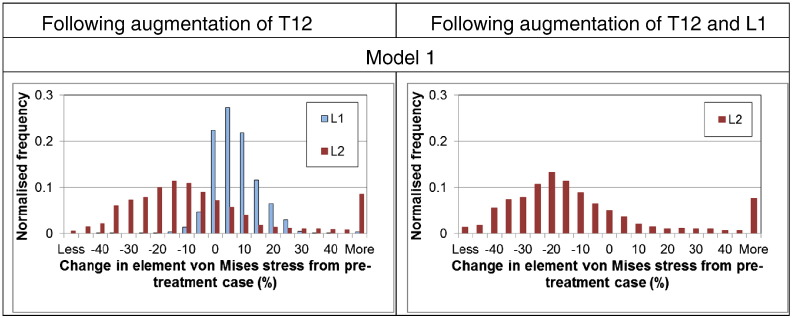
Distribution of change in the von Mises stress in the elements of the L1 and L2 vertebrae following treatment. The change is calculated as a percentage difference in stress in each element compared to the same element in the untreated model. Normalising is by the total number of elements in the corresponding vertebra.

The von Mises stress distribution through the vertebrae was compared between the pre-treatment case and those following augmentation of T12 and both T12 and L1, as shown in Fig. 4. The changes in the von Mises stress in each bone element between the untreated and treated cases are shown in histogram form in Fig. 5. When T12 was augmented, there were large changes in the stress distribution in both L1 and L2. More elements in L1 underwent an increase in von Mises stress, whereas in L2 the stress decreased in majority of elements. For L2 in particular, there was greater load being carried through the central region and correspondingly less through the cortex following augmentation. When both T12 and L1 were augmented, there was little further alteration in the stress distribution compared to the case when only T12 was augmented.

## 4 Discussion

The aim of this study was to use a combined computational and experimental approach to assess the biomechanical effects arising from vertebroplasty, in relation to a combination of patient factors including bone and disc quality, and treatment variables such as the cement volume.

The FE models in this study were developed from CT scans of experimental specimens. In addition to extracting the geometry from the scans, the hard tissue properties were also derived from the image greyscale on an element-by-element basis. Currently, however, there is no methodology available to obtain specimen-specific intervertebral disc material data from these images. Hence, a reverse engineering approach was used in which the disc material properties were tuned so that the model stiffness matched the experimentally measured pre-augmentation elastic behaviour. The post-augmentation predictions of this model as well as the second model independent of the tuning process were then compared with respective experimental test data. In both cases, the good agreement indicated that the models were able to capture the mechanical behaviour and the changes that occur due to the augmentation. This approach could now be applied to more specimens to build greater confidence in the model predictions over a larger dataset. Since at this stage, the validation was only undertaken on one independent specimen, and the stress measurements were not validated experimentally, the stress values were not discussed in terms of their magnitudes but compared in terms of relative differences between the parametric studies.

The gross behaviour of the spinal segments is influenced most by the lowest stiffness components, that is, by the intervertebral discs. This can be seen in Fig. 3 where the difference between the healthy and degenerated disc models is large. The bone quality also plays a role, particularly with degenerated discs where the difference in elastic behaviour between bone and disc components is lower. Superimposed onto these trends is the stiffening effect of the cement, with larger volumes of cement increasing the segment stiffness. This result is similar to conclusions outlined by Molloy et al. (2003). In current clinical practice different cements are used for the augmentation. However, the parametric variation of the cement modulus indicated little influence on the overall segmental stiffness, as well as on the change in elemental stress in the adjoining non-augmented vertebrae. The cement volume has the greatest influence on the stiffness in the degenerated disc models that exhibit good bone quality. However, similar to observations made by Graham et al. (2007), when the bone quality is poor, increasing cement volume has only a weak contribution towards stiffness improvement. From a clinical point of view, it is patients with low BMD who would be flagged as being at risk of vertebral fracture and who could, therefore, benefit from a prophylactic vertebroplasty. This study indicates that the disc quality will influence the change in segmental stiffness due to cement augmentation. One of the key objectives of vertebroplasty is to restore the vertebral stiffness. However, a much higher stiffness at a particular level in the spine compared to the adjoining levels is not an ideal scenario.

The segmental stiffness gives some indication of the overall effect of the cement augmentation. However, it is the risk that the procedure changes the load distribution through the segment that has been highlighted in previous studies (Baroud et al., 2003; Polikeit et al., 2003; Wilcox, 2006), particularly with respect to adjacent vertebral fracture. The FE analysis was performed to replicate the experiments conducted under displacement control. In-vivo conditions generally represent a load control situation. Results in Figs. 4 and 5 were therefore obtained at a common reaction force of 1000 N to allow comparison of results at the same load level. It can be observed from these figures that when T12 vertebra is augmented, there is a change in the stress distributions in both L1 and L2. Perhaps, more significantly, the majority of elements in the adjoining vertebra experienced an increase in von Mises stress. This load redistribution following augmentation would be a matter for concern, especially if the bone quality in the adjoining vertebra is poorer. In contrast to L1, the L2 vertebra exhibited a relative reduction in von Mises stress for the majority of elements. Since it is connected to the fixed boundary conditions (at the bottom), the biomechanical effects relevant to in-vivo physiological conditions cannot be concluded from this model. The position of the applied load in the models replicated the same anatomical position in the experiments, but in reality, the load will change with activities, so this result only provides one snapshot of the daily range of loading on the segment. This result also has implications for experimental testing and suggests that care needs to be taken in making comparisons between specimens because the loading position and boundary conditions may influence the behaviour. Further loading scenarios and a greater number of model morphologies now need to be investigated, not only under displacement control conditions but also under load-control, to gain greater understanding of the influence of the load and vertebral shape on the outcomes of vertebroplasty.

## 5 Conclusion

The results of this study demonstrate that in addition to the cement volume, patient variables such as bone and disc quality and spinal morphology all influence the mechanical effect of vertebroplasty. This starts to indicate why there may be very different outcomes in vertebroplasty from one patient to another. A much greater number of model morphologies and range of loading conditions now need to be considered to enable conclusions to be drawn as to how the material and morphological characteristics of the spine could be used to stratify the patients and dictate different treatments. The specimen-specific FE approach presented here provides a potential route to achieve this and these methods could now be applied to a larger specimen cohort.

## Figures and Tables

**Table 1:** Material properties for the components used in the finite element model.

Material	Elastic modulus (MPa)	Poisson's ratio	Reference
Bone	Element-specific from CT data Mean:128.41, Standard Deviation: 66.20	0.3	Jones and Wilcox (2007) and Wijayathunga et al. (2008)
Cement housing	2450	0.3	McCormack et al. (1999)
Injected cement	2040	0.3	Jasper et al. (2002) and Oakland et al. (2009)
Nucleus pulposus: Healthy	1	0.499	Grauer et al. (2006)
Degenerated	4.9	0.43	
Annulus fibrosus^a^ healthy	E_1_ = 0.2, E_2_ = 35, E_3_ = 8	ν_12_ = 0.02, ν_13_ = 0.065, ν_23_ = 1.2	Elliott and Setton (2000)
Degenerated	E1 = 0.53, E2 = 91.9, E3 = 21	ν_12_ = 0.022, ν_13_ = 0.072, ν_23_ = 1.32	
Capsular ligament	10	0.3	Grauer et al. (2006)

a Directions 1, 2, 3 are radial, circumferential and axial respectively.
